# Efficacy and safety of pediatric massage in the treatment of anorexia: A protocol for systematic review and meta-analysis

**DOI:** 10.1097/MD.0000000000025654

**Published:** 2021-04-30

**Authors:** Baoxiu Yi, Wenguang Chen, Gen Deng, Yiyi Wang, Jinfeng Wang, Zhenhai Chi

**Affiliations:** a College of Acupuncture and Massage, Jiangxi University of Traditional Chinese Medicine; b Affiliated Hospital of Jiangxi University of Traditional Chinese Medicine, Nanchang, China.

**Keywords:** anorexia, pediatric massage, protocol, systematic review and meta-analysis, Traditional Chinese Medicine therapy

## Abstract

**Background:**

Anorexia is a common and frequent disease in clinical pediatrics. It refers to a chronic digestive disorder syndrome with loss of appetite or disappearance and reduced food intake. The incidence of anorexia in children is very high, if not timely, safe and effective treatment, will have a huge impact on the growth and development of children. The toxic side effects of related treatment drugs often worry clinicians. Pediatric massage is external therapy, with green, safe and effective characteristics, lack of evidence-based medicine evidence support. A systematic evaluation and meta-analysis of the safety and efficacy of pediatric massage in the treatment of anorexia will be carried out in this paper to provide a powerful evidence.

**Methods:**

We’ll retrieve 8 electronic databases, including the PubMed, Embase, Cochrane Library, Web Of Science, Chinese Biomedical Literature Database (CBM), China National Knowledge Infrastructure (CNKI), Wanfang Database (WF), China Science Journal Database (VIP), the retrieval date was established from the database to March 2021. The authors will screen the study independently, Extracting data, and through Coch bias tools to assess the quality hazards of methods. RevmanV.5.3 software will be used for statistical analysis.

**Results:**

The results of this study need to be obtained after the completion of this program.

**Conclusions:**

The conclusion of this study will confirm the efficacy and safety of pediatric massage in the treatment of anorexia, and provide reliable evidence for clinical selection of pediatric massage in the treatment of anorexia.

**Ethics and dissemination:**

This study does not need to be reviewed by the Ethics Committee, because this paper is not a clinical study or a related experimental study, and this paper is only a literature study.

**INPLASY Registration number:**

INPLASY202130050.

## 1 Introduction

Anorexia is a common and frequently-occurring disease in clinical pediatrics. It refers to a chronic digestive disorder syndrome in which appetite decreases or disappears and appetite is reduced.^[1]^ Epidemiological surveys abroad show that the incidence of aversion to eating in infants and preschool children is 12% to 34%.^[2]^ Children with anorexia in children will show gastrointestinal discomfort, abdominal pain, abdominal swelling, vomiting, constipation, and anorexia. The appearance of these symptoms can indicate to a certain extent that the child's digestive tract system has organic disease,^[3]^ Anorexia in children can lead to malnutrition. Long-term anorexia will affect the growth and development of children and reduce immunity, which will lead to a variety of diseases. In severe cases, children will develop infections of the central system or mental disorders.^[4]^ At present, the treatment of anorexia in children in modern medicine is mainly based on conventional drugs, such as the application of drugs that promote gastrointestinal motility, gastrointestinal biological agents, etc. These drugs can improve the symptoms of children to a certain extent, but a large number of clinical reports found that this type of drug has relatively large side effects, which is not conducive to the physical and mental health of patients and children. Pediatric tuina is an external Traditional Chinese Medicine treatment method. It does not require injections or medications. It acts on the meridians and acupoints of the human body to treat anorexia. It is safe, non-toxic, green, and effective. In view of more and more clinical reports, it is said that massage in children has a significant effect on the treatment of anorexia,^[5–7]^ but there is no relevant evidence-based medical evidence to confirm it. Therefore, this study will conduct a systematic review and meta-analysis of the effectiveness and safety of pediatric massage for the treatment of anorexia.

## 2 Methods

### 2.1 Study registration

This protocol was registered with the International Platform of Registered Systematic Review and Meta-Analysis Protocols (INPLASY) on March 15, 2021 and was last updated on March 15, 2021 (registration number INPLASY202130050).

### 2.2 Inclusion criteria for study selection

#### 2.2.1 Types of studies

Clinical randomized controlled trials (RCT) containing pediatric massage for anorexia will be included, but do not limit language and publication status.

#### 2.2.2 Types of participants

There are clear and recognized diagnostic and curative criteria, and all patients are diagnosed with anorexia in children, regardless of sex, age, and source of cases.

#### 2.2.3 Types of interventions

##### 2.2.3.1 Experimental interventions

Pediatric massage will include all different schools of pediatric massage techniques. Mixed therapy based on pediatric massage will also be included.

##### 2.2.3.2 Control interventions

The control group will receive one of the following treatments: routine pharmaceutical treatment, no treatment, and placebo.

#### 2.2.4 Types of outcome measures

##### 2.2.4.1 Primary outcome

Clinical efficacy, including total effective rate or cure rate, clinical symptom score, will be considered as the primary outcome.

##### 2.2.4.2 Secondary outcomes

Body mass index (BMI), Traditional Chinese Medicine symptom score changes, and recurrence will be secondary outcomes.

### 2.3 Exclusion criteria

Non-randomized controlled trials; no exact diagnostic scale or therapeutic scale; no moxibustion as the main treatment in the experimental group, and moxibustion therapy was found in the control group. Repeated literature; theory and review literature; animal experiments; nursing research.

### 2.4 The retrieval methods and strategies of this study

#### 2.4.1 Electronic database retrieval

We’ll retrieve 8 databases, the electronic databases, including the PubMed, Embase, Cochrane Library, Web Of Science, Chinese Biomedical Literature Database (CBM), China National Knowledge Infrastructure (CNKI), Wanfang Database (WF), China Science Journal Database (VIP), The retrieval date was established from the database to March 2021, without any language restriction. And will searching the relevant literature by combining subject words with free words, search terms consist (“children” or “pediatric” or “baby” or “infant” or “minors”) AND (“anorexia” or “cibophobia” or “Anorexia nervosa” or “piddle”) and intervention (“Massage” or “Tuina” or “manipulative therapy”) and research types (“randomized controlled trial” or “controlled clinical trial” or “random trials” or “RCT”). The PubMed search strategy is shown in Table 1.

**Table 1:** Retrieval strategies in PubMed.

ID	Query
#1	“Pediatric”[Mesh]
#2	(((children[Title/Abstract]) OR (baby[Title/Abstract])) OR (infant[Title/Abstract])) OR (minors[Title/Abstract])
#3	#1 OR #2
#4	“anorexia”[Mesh]
#5	((cibophobia[Title/Abstract]) OR (Anorexia nervosa[Title/Abstract])) OR (piddle[Title/Abstract])
#6	#4 OR #5
#7	“Massage”[Mesh]
#8	(Tuina[Title/Abstract]) OR (manipulative therapy[Title/Abstract])
#9	#7 OR #8
#10	(((randomized controlled trial[Ti/Ab]) OR (random trials[Ti/Ab])) OR (controlled clinical trial[Ti/Ab])) OR (RCT[Ti/Ab])
#11	#3 AND #6 AND #9 AND #10

#### 2.4.2 Searching other resources

We will combine manual retrieval of literature resource database to search relevant conference papers that meet the inclusion criteria. In addition, the grey literature, as well as ongoing and recently completed studies, will be searched on Clinicaltrials.gov.

### 2.5 Data extraction and management

#### 2.5.1 Literature inclusion and data extraction

**Figure 1:**
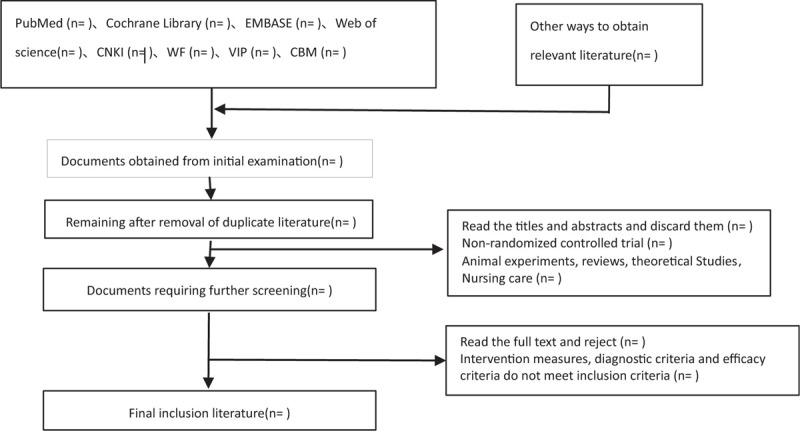
Flow chart of literature incorporation.

Two reviewers will independently extract relevant data from the eligible RCTs, including the first author, participants’ baseline characteristics, sample size, intervention, intervention time, follow-up, results, and adverse events. Any discrepancies will be resolved through consultation with a third reviewer. If necessary, we will also contact the original author for more information. The inclusion process of this study will be carried out as shown in Fig. 1.

#### 2.5.2 Methodological quality evaluation

Two evaluators independently select the literature according to the inclusion and exclusion criteria and cross-check. In case of disagreement, a third evaluator will assist in the decision. The extracted data included the first author, year of publication, number of patients, age, sex, intervention measures, outcome indicators, etc. The Jadad scale to evaluate quality into literature, including: random sequence (right 2 points, 1 points not clear, inappropriate 0), distribution, hidden (right 2 points, 1 points not clear, inappropriate 0), blinded (right 2 points, 1 points not clear, inappropriate 0), lost to follow-up and exit (describe 1 points, not describe 0); 0–3 is classified as low quality and 4–7 as high quality.

### 2.6 Quantitative data synthesis and statistical methods

#### 2.6.1 Quantitative data synthesis

We will use RevMan V.5.3 software for statistical analysis. For continuous variables, when outcomes were measured by the same scale, the results were reported as standardized mean difference (MD) and 95% confidence interval (CI); when different scales were used, the results were reported as standardized mean difference (SMD) and 95% CI. Categorical data will be calculated with the risk ratio (RR) and 95% CI.

#### 2.6.2 Assessment of heterogeneity

We will use *I*^2^ test and Chi-square test to evaluate the heterogeneity of the results. When *I*^2^ ≤ 50% and *P* > .10, the results of the study will be considered as homogeneous, and fixed effect model will be used; otherwise, random effect model will be used.

#### 2.6.3 Subgroup analysis

If significant heterogeneity is detected in our meta-analysis, we will perform subgroup analysis based on different control groups.

#### 2.6.4 Sensitivity analysis

When there are sufficient RCTs, we will conduct sensitivity analysis based on methodological quality, sample size, and missing data to evaluate the robustness of the research results.

#### 2.6.5 Assessment of reporting biases

Publication bias will be analyzed through the funnel plot. If the funnel plot is asymmetric, there may be a publication bias in the research results.

## 3 Discussion

Anorexia in children is a common and frequently-occurring disease in children characterized by aversion to eating for a long time, poor appetite, or reduced appetite.^[8]^ The clinical manifestations are loss of appetite and food intake below the normal level by >60%. Some children may refuse to eat.^[9]^ If children's appetite is not improved for a long time, it will cause malnutrition, anemia, weight loss, affect growth and development, and cause severe impact on children's immunity and intelligence, as well as increase susceptibility to other system diseases.^[10]^ The pediatric massage of traditional Chinese medicine can reconcile qi and blood, adjust yin and yang, effectively improve the patient's spleen and stomach weakness, alleviate the patient's indigestion, and can completely cure the disease.^[11]^ At present, more and more studies believe that pediatric massage has a good clinical effect in the treatment of anorexia, and the clinical acceptance is high, but the author has not yet seen a systematic review of the effectiveness and safety of related pediatric massage in anorexia. Therefore, this article needs to systematically evaluate and meta-analyze the safety and effectiveness of Tuina therapy in children with anorexia, so as to provide evidence-based medical evidence for future clinical guidance.

## Author contributions

**Data curation:** Baoxiu Yi, Wenguang Chen.

**Formal analysis:** Baoxiu Yi, Gen Deng.

**Investigation:** Baoxiu Yi, Wenguang Chen.

**Methodology:** Wenguang Chen, Yiyi Wang.

**Project administration:** Jinfeng Wang, Zhenhai Chi.

**Software:** Gen Deng, Jinfeng Wang.

**Supervision:** Baoxiu Yi, Zhenhai Chi.

**Validation:** Gen Deng, Zhenhai Chi.

**Visualization:** Yiyi Wang, Jinfeng Wang.

**Writing – original draft:** Baoxiu Yi, Zhenhai Chi.

**Writing – review & editing:** Baoxiu Yi, Zhenhai Chi.
